# Noninvasive Techniques for Management of Erythema Multiforme

**DOI:** 10.1155/2023/9938939

**Published:** 2023-12-19

**Authors:** Fabiana Martins, Debora Pallos, Jodkandlys Candeia, Rodrigo Zerbinati, Paulo Henrique Braz-Silva, Luana Campos

**Affiliations:** ^1^Postgraduate Program in Dentistry, School of Dentistry, University of Santo Amaro, Rua Prof. Enéas de Siqueira Neto, 340-Jardim das Imbuias, São Paulo, SP 04829-300, Brazil; ^2^Department of Stomatology, School of Dentistry, University of São Paulo, Av. Prof. Lineu Prestes, 2227, São Paulo, SP 05508-000, Brazil; ^3^Laboratory of Virology (LIM-52), Institute of Tropical Medicine of São Paulo, School of Medicine, University of São Paulo, Rua Prof. Enéas Carvalho de Aguiar, 470-Cerqueira Cesar, São Paulo, SP 05403-000, Brazil

## Abstract

An 18-year-old man was referred for a diagnosis of extensive oral lesions. During the interview, he reported a medical history of ganglionic tuberculosis, type 2 herpes infection, and significant weight loss due to dysphagia. Intraoral exam revealed multiple painful and ulcerated lesions covered by pseudomembrane. Lesions were observed on the labial and buccal mucosa, tongue, and soft palate. The laboratory findings included serum positivity for the Epstein-Barr virus, and salivary tests showed positive values for herpes simplex virus (HSV-2) and human herpesvirus (HHV-7). The diagnostic hypothesis was based on clinical findings and viral infection detected in the saliva, which triggered an immunological disorder, that is, erythema multiforme (EM). The treatment consisted of antimicrobial photodynamic therapy (aPDT), with substantial improvement in pain and healing as seen in the following twenty-four hours. Complete resolution of the lesions was achieved five days after the first session. Once the diagnosis of virus-induced EM was confirmed, noninvasive techniques (e.g., salivary tests and aPDT) were very successful and can be indicated for managing these lesions.

## 1. Introduction

Erythema multiforme (EM) is a severe inflammatory mucocutaneous reaction which presents with multiple, extensive, and coalescent ulcers. Skin lesions are common but not always present in all cases of EM. In almost 50% of the cases, EM occurs from the increase of infections, such as herpesviruses, mycoplasma pneumonia, hepatitis C virus, Coxsackie virus, or medications [1, 2]. Clinically, EM is characterized by multiple, extensive, and painful ulcers which coalesce, thus affecting the oral cavity. Skin lesions can be seen on the palms of the hands, often called target lesions, but they are not always present in all cases of EM [1].

In general, the treatment consists of removing the causative agent, either it is biological or infectious [2, 3]. The use of steroids is controversial as they are not always considered to be effective. In cases where there is an association with viral infections, the use of antivirals is recommended [2]. Nevertheless, in cases where the signs and symptoms are severe, the concomitant use of alternative therapies has shown to produce promising results.

There are still limited reports regarding the use of lasers as an alternative treatment for EM. Although its analgesic, biomodulating, and anti-inflammatory effects have already been proven, including an antimicrobial effect when photodynamic therapy is performed, its benefits are inconclusive for EM. Therefore, we described the clinical use of local noninvasive therapy, including a daily association of photobiomodulation therapy (PBMT) and antimicrobial photodynamic therapy (aPDT), which resulted in a good prognosis.

## 2. Case Presentation

**Figure 1:**
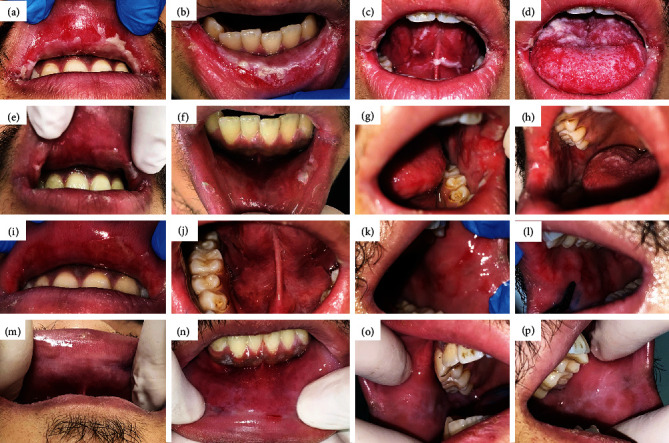
Clinical course. (a–d) Initial oral examination of the oral lesions. (e–h) After 24 hours from the first aPDT and PBMT sessions. (i–m) Clinical aspect after 48 hours. (n–p) 5^th^ day after laser therapy.

An 18-year-old man was referred to an oral medicine clinic with a chief complaint of painful and nonhealing multiple oral ulcerations, malaise, and weight loss (2.2 pounds) due to dysphagia. Medical history revealed a recurrence of ganglionic tuberculosis, which had been diagnosed five years before. The patient has been using medications (antimicrobial therapy) for 15 days, including isoniazid (300 mg), ethambutol (1000 mg), pyrazinamide (1000 mg), and rifampicin (600 mg). In addition, the patient reported being a smoker (1 pack per day for 1 year). Hematological and serological exams showed increased values of C-reactive protein (CRP) (13.9 mg/L), positivity for the Epstein-Barr virus (EBV-Anti − VCA − IgM > 160 U/mL) and negativity for syphilis, HIV, and cytomegalovirus. During the oral examination, the patient presented with confluent multiple ulcerations with erythematous halo covered by pseudomembrane distributed on the upper and lower lips, bilateral buccal mucosa, tongue, soft palate, and oral pharynx (Figures 1(a)–1(d)). Medical history and physical examination led to the following possible diagnoses: oral tuberculosis ulcers, drug-induced ulcerations, and erythema multiforme.

As the tests were positive for herpes simplex virus 2 (HSV-2) and human herpesvirus 7 (HHV-7), the diagnostic hypothesis of herpetic stomatitis associated with EM was made. Due to the pattern of oral lesions and detection of HHV infection confirmed in blood and saliva tests, the diagnosis of minor type of EM was established.

Because the use of analgesics was ineffective in controlling pain and due to the severity of oral lesions, there was an indication of topical therapy, which included the daily association of photobiomodulation therapy (PBMT) and antimicrobial photodynamic therapy (aPDT). No other medications were prescribed.

For PBMT, a low-level laser device (Therapy EC®, DMC, São Carlos, SP, Brazil) was used on contact mode and point by point, initially at 660 nm with 100 mW, 35 J/cm^2^, 1 J, and 10 sec per point. A total of 80 points were distributed throughout all lesions. Next, PBMT was performed only in painful lesions following the same protocol (1 J of energy), but at 808 nm. Finally, aPDT technique was performed with methylene blue 0.01% applied over the lesions. After three minutes (preirradiation time), the same laser device was used at 660 nm, 5 J, and 50 sec per point for one point per lesion, which corresponded to 1 hour of clinical procedure. Within 24 hours, there was a reduction in lesion severity and pain (10 to 5, according to the visual analogue scale (VAS)) (Figures 1(e)–1(h)). Within 48 hours, the patient resumed eating solid foods (Figures 1(i)–1(l)), mucosal healing was achieved after the fifth day since the first laser session, and all normal oral functions were restored (Figures 1(m)–1(p)). Due to the good clinical response to PBMT and aPDT, additional complementary exams were not requested.

## 3. Discussion

Erythema multiforme (EM) is an ulcerative-bullous lesion probably resulting from an immunological cause occurring in the oral mucosa, with or without wounds in the skin [4]. The etiopathogenesis of EM includes infections such as herpesviruses (HHV simplex and EBV) or other pathogens such as mycoplasma pneumonia, hepatitis C virus, Coxsackie virus, and COVID-19 or its RNA vaccines, including the use of medications, mainly antibiotics or analgesics [5, 6].

EM is usually seen in young male adults who present the following clinical signs: fever, malaise, headache, cough, and sore throat, which precede the appearance of visible lesions. EM can be categorized as minor or major, followed by diffuse desquamation and ulceration on the entire skin surface and mucous membranes. Major EM differs from other types of severe vesicle-bullous dermatological disorders such as the Stevens-Johnson syndrome (SJS) and toxic epidermal necrolysis (TEN), which are more associated with the use of medications [5–7].

Clinically, EM has an abrupt onset and presents shallow ulcers, sometimes coalescing and exhibiting irregular contours, which may exhibit hemorrhagic crusting and swelling. These lesions are diffusely distributed in the oral cavity, mostly affecting lips, labial mucosa, buccal mucosa, and dorsum of the tongue. The treatment of EM can be challenging as the literature recommends the use of antivirals, antibiotics, and corticosteroids, meaning that the most adequate therapy will be indicated once the possible etiological agent has been determined [7, 8].

Due to their severity, the EM lesions are painful and usually make it impossible to maintain proper oral functions [4]. Therefore, regardless of the mucocutaneous involvement or severity of signs and symptoms, medical history and infections should be investigated once EM has been suspected. In the present case, the patient had a history of recurrence of HHV-1 and HHV-2, and at the time of sessions, recent blood tests showed viremia for EBV. Given the oral clinical presentation of the lesions and medical history, a diagnostic hypothesis of EM associated with herpes infection was made.

Saliva samples were positive for HHV-2 and HHV-7, thus being a plausible alternative approach due to the patient's reduced mouth opening and presence of pain. In fact, this diagnostic tool prevented the performance of an incisional biopsy. This case illustrates the importance of including salivary tests in the differential diagnosis of oral lesions, contributing to the determination of more effective treatments and better prognosis, especially in an immunocompromised patient.

Reactivation of infections, especially by those viruses belonging to the herpesvirus family, is common in immunocompromised patients. These opportunistic infections can worsen local or systemic conditions in these patients. Additionally, these infections can accumulate and lead to many scenarios involving painful oral lesions that are present, such as grades 3-4 oral mucositis and graft-versus-host disease [9].

PBMT modulates substances, which play essential roles in the pain and inflammatory modulation, and aPDT is highly effective in managing opportunistic infections of the oral cavity, including virucidal activity [10]. In addition, the techniques are simple and effective in promoting the healing of severe wounds and in relieving the pain, which is essential for restoring oral functions, with side effects only seen when incorrect [11, 12]. Therefore, aPDT can be considered as a safe technique with rare adverse events when photosensitizers are used at incorrect concentration.

The literature presents promising results for the use of aPDT technique as a treatment of herpesvirus lesions, which is based on virus inactivation (i.e., fragmentation of viral DNA) by the oxidative potential of the photosensitizers after light absorption, without any side effect [9, 10, 13].

Our results are also in accordance with other clinical findings on the efficacy of PBMT in treating severe cases of EM as it has been effective in minimizing the duration of signs and symptoms. Recent case reports showed that pediatric patients diagnosed with SJS and daily treated with PBMT had an accelerated restoration of oral functions, including analgesia and improved quality of life [13–16].

Although the biological mechanisms underlying the therapeutic effects of PBMT have not been completely elucidated, clinical and laboratory studies suggest that the absorption of laser light by intracellular mitochondrial cytochrome c-oxidase promotes an increase in ATP production [17]. In this process, a series of intracellular signals are also activated (e.g., reactive oxygen species/EROS, cAMP, and NF-*κ*B), which induce gene transcription of essential proteins for tissue repair. In addition, the balance between prooxidant and antioxidant mediators is restored, thus reducing oxidative stress [17–19].

As for the effect of light-induced analgesia, several theories are described in the literature. The best-known theory is the direct action of laser light on photosensitive ion channels in the plasma membrane of nerve terminals, such as the transient receptor potential channels [20]. In this context, the use of a local noninvasive dental approach for an immunocompromised patient was assertive in rapidly causing remission of the lesions, which was already noticeable after the first laser irradiation.

## 4. Conclusion

The presence of oral lesions in immunocompromised patients represents a clinical challenge for clinicians. Therefore, the evaluation of laboratory exams, associated with a detailed intraoral examination, is fundamental for establishing the diagnosis and indicating an adequate management of the observed alterations. Saliva test and the proposed local treatment were well accepted by the patient and did not interact with the drug therapy used, thus promoting rapid resolution of the lesions.

## Data Availability

The clinical data used to support the findings of this study are included within the article.
